# Editorial: Overcoming resistance in DDR inhibition: new targets and therapeutic strategies

**DOI:** 10.3389/fmolb.2025.1766744

**Published:** 2026-01-05

**Authors:** Chi-Lin Tsai, Ana Cristina Gonçalves, Zamal Ahmed

**Affiliations:** 1 Molecular Oncology, University of Texas at MD Anderson Cancer Center, Houston, TX, United States; 2 Faculty of Medicine (FMUC), Coimbra Institute for Clinical and Biomedical Research (iCBR), Area of Environment, Genetics and Oncobiology (CIMAGO), University of Coimbra, Coimbra, Portugal

**Keywords:** cancer therapy, DDR, DNA repair, drug discovery, PARP inhibitor, therapeutic resistance

The killing of the malignant tumors with minimal effect on normal tissues is the most desirable outcome for cancer treatment. Yet, chemo- and radiation therapies that cause irreparable DNA damage are still the most successful first-in-line treatments for most cancers. While these therapies do kill cancer cells, they can also have an impact on healthy cells manifesting as toxic side effects and ultimately resistance. As a result, researchers are continually exploring new therapeutic options to improve efficacy and minimize patients’ discomfort. Poly ADP-ribose polymerase (PARP) inhibitors are being developed to target DNA repair pathways in patients with genetic mutations in the BRCA1 or BRCA2 proteins. PARP inhibitors work by blocking the catalytic activity (PARylation) of PARP enzymes, which repair single-stranded DNA breaks (SSBs). In patients with BRCA mutation, this impairs the repair of double-stranded DNA breaks (DSBs), leading to a phenomenon known as synthetic lethality that ultimately results in the killing of cancer cells. The FDA approved the first PARP inhibitor, olaparib, in 2014 to treat patients with germline *BRCA* mutations for ovarian, breast, and pancreatic cancers. The PARP inhibitor, which employs the synthetic lethality strategy, is effective but also has limitations. Between 40% and 70% of patients treated with PARP inhibitors develop resistance (D'Andrea, 2018; Li et al., 2020; Zou et al., 2025). Therapeutic resistance in DDR frequently activates alternative or backup DNA repair pathways or bypasses conventional cell cycle checkpoints to tolerate DNA damage. PARP inhibitor treatment of homologous recombination (HR)-deficient BRCA-mutation cancer cells, for example, can activate alternative end joining (Alt-EJ) to repair DSBs or restore HR function through reversion of BRCA mutations or loss of non-homologous end-joining (NHEJ) regulators such as 53BP1 or REV7 (Jackson and Moldovan, 2022). This resistance presents a significant therapeutic challenge and motivates us to initiate this Research Topic to collect new cancer targets and therapeutic strategies to overcome resistance to DNA damage response (DDR) inhibition.

This Research Topic encompasses various aspects, including inhibitor discovery, therapeutic strategies, and the molecular mechanisms involved in DNA damage response and repair proteins. For example, cancer heterogeneity and rapid mutation rates causing therapeutic resistance present a significant medical challenge. With improved computational speed thanks to advanced graphics processing unit (GPU) development, *in silico* screening of small molecules guided by artificial intelligence-machine learning (AI-ML) becomes more efficient and may accelerate drug discovery. Moiani and Tainer developed a Goldilocks computational protocol to target DDR proteins to efficiently identify lead compounds for DDR targets using a virtual screen protocol. Their protocol allows efficient lead identification that can be fast tracked into early compound bound protein structures and structure guided drug design for generating lead which overcome bottlenecks that can limit timely breakthrough drug discoveries. Several DDR targets have been practiced using this protocol and will be validated with experimental data.

Immunotherapies target specific immune checkpoints, such as blocking the PD-1 and PD-L1 interaction, allowing T cells to attack cancer cells. However, resistance can develop over time for cancers with low PD-L1 expression or alterations in the tumor microenvironment. Thus, combination cancer therapy that includes immunotherapy and other cancer treatments may result in better patient outcomes. Lin et al. established a radiosensitivity index model using 10 DNA repair-related genes, including ARTEMIS, RECQL4, H2AX, and GTF2H5, to predict radiotherapy benefit in breast cancer. Their findings indicate a strong correlation between radiosensitivity, immunity, and immunotherapy efficacy, especially in radiosensitive and high PD-L1 BRCA patients. Similarly, Dagar et al. discussed the therapeutic strategy of combining radiotherapy and immunotherapy (radioimmunotherapy). They summarized the DDR antagonists’ effects on intracellular immunological responses during radioimmunotherapy and highlighted clinical practices, outcomes, and toxicity profiles when adopting this strategy. Thus, a better understanding of the interplay between DDR and tumor immunity could improve our capacity to synergistically integrate immunotherapy with other cancer therapies.

The discovery and deeper understanding of DDR targets and mechanisms may lead to more effective therapeutic strategies and improved drug targeting sites, hence reducing resistance. This could potentially improve patient outcomes while reducing the adverse effects associated with standard treatments. Alphey et al. reported a deeper understanding of how sliding clamp PCNA binding proteins (DNA polymerase Pol δ, the translesion DNA synthesis polymerase Pol ζ, and FEN1) interact with PCNA via its PCNA-interacting protein (PIP) motif both within and across species. The canonical binding modes of FEN1 PIP peptide-PCNA and PolD3 PIP peptide-PCNA from *Chaetomium thermophilum* were identified using biophysical binding studies and X-ray crystallography. These additional structural insights contributed to a more complete understanding of the evolution of PCNA-PIP interactions, providing further information for inhibitor development. Furthermore, combining high-resolution structures with evolutionary analyses could enhance our understanding of the impact of cancer mutations, particularly variants of unknown significance (VUS). The quantitative assessment of VUS scoring in the context of DDR nucleases and helicases demonstrated the ability to identify cancer mutations with important functional consequences (Tsutakawa et al., 2021).

These articles broaden our knowledge of DDR proteins and contribute substantially to therapeutic strategies. A deeper comprehension of DDR protein crosstalk among DNA repair pathways and other cellular processes such as metabolism, inflammation, or cell cycle checkpoints; the discovery of novel DDR protein-protein interactions through cellular, functional, and structural studies; and the association between DDR deficiencies and clinical outcomes as well as therapeutic responses may enhance the formulation of more targeted therapies and combined therapies to improve cancer patient outcomes.
